# Challenges in the diagnosis of neurofibromatosis type 1 (NF1) in young children facilitated by means of revised diagnostic criteria including genetic testing for pathogenic *NF1* gene variants

**DOI:** 10.1007/s00439-021-02410-z

**Published:** 2021-12-20

**Authors:** Hildegard Kehrer-Sawatzki, David N. Cooper

**Affiliations:** 1 Institute of Human Genetics, University Hospital Ulm, University of Ulm, Albert-Einstein-Allee 11, 89081 Ulm, Germany; 2 Institute of Medical Genetics, Cardiff University, Heath Park, Cardiff, CF14 4XN UK

## Abstract

Neurofibromatosis type 1 (NF1) is the most frequent disorder associated with multiple café-au-lait macules (CALM) which may either be present at birth or appear during the first year of life. Other NF1-associated features such as skin-fold freckling and Lisch nodules occur later during childhood whereas dermal neurofibromas are rare in young children and usually only arise during early adulthood. The NIH clinical diagnostic criteria for NF1, established in 1988, include the most common NF1-associated features. Since many of these features are age-dependent, arriving at a definitive diagnosis of NF1 by employing these criteria may not be possible in infancy if CALM are the only clinical feature evident. Indeed, approximately 46% of patients who are diagnosed with NF1 later in life do not meet the NIH diagnostic criteria by the age of 1 year. Further, the 1988 diagnostic criteria for NF1 are not specific enough to distinguish NF1 from other related disorders such as Legius syndrome. In this review, we outline the challenges faced in diagnosing NF1 in young children, and evaluate the utility of the recently revised (2021) diagnostic criteria for NF1, which include the presence of pathogenic variants in the *NF1* gene and choroidal anomalies, for achieving an early and accurate diagnosis.

## Introduction

Neurofibromatosis type 1 (NF1, MIM#162200) is an autosomal dominant inherited genodermatosis and tumour predisposition syndrome with an incidence of 1:3000 (Lammert et al. 2005). NF1 is caused by pathogenic variants in the *NF1* gene on chromosome 17q11.2 and characterized by skin pigmentation anomalies such as café-au-lait macules (CALM) and skin-fold freckling, as well as dermal neurofibromas. Additionally, NF1 patients frequently have Lisch nodules, learning disabilities, attention deficits, hyperactivity, skeletal abnormalities, plexiform neurofibromas and optic pathway gliomas. The criteria for the diagnosis of NF1 were established by a National Institutes of Health (NIH) Consensus Conference in 1988 (Neurofibromatosis: conference statement: National Institutes of Health Consensus Development Conference 1988). They comprise the most common clinical features observed in NF1 (Table 1). Two or more of the 7 listed criteria must be met in a given individual for a definitive diagnosis of NF1 to be made. These diagnostic criteria have been widely used in the clinical routine since their original formulation in 1988. However, the discovery of other genetic disorders with clinical symptoms overlapping those of NF1 made the revision of these criteria necessary (Legius et al. 2021). The diagnostic criteria were also revised to facilitate the diagnosis of NF1 in young children who present with isolated CALM but no other disease manifestations or a family history of NF1 and hence do not meet the strict 1988 clinical diagnostic criteria. The revised diagnostic criteria for NF1 were published recently as an international consensus recommendation (Table 1) (Legius et al. 2021). They include the detection of a pathogenic variant in the *NF1* gene as a diagnostic criterion which allows for an early diagnosis of NF1 in oligosymptomatic children without a family history of the disease. The revised diagnostic criteria for NF1 also include choroidal anomalies as a new ophthalmic symptom with high sensitivity and specificity for NF1 which may facilitate the diagnosis of NF1 particularly in young oligosymptomatic children.

**Table 1:**
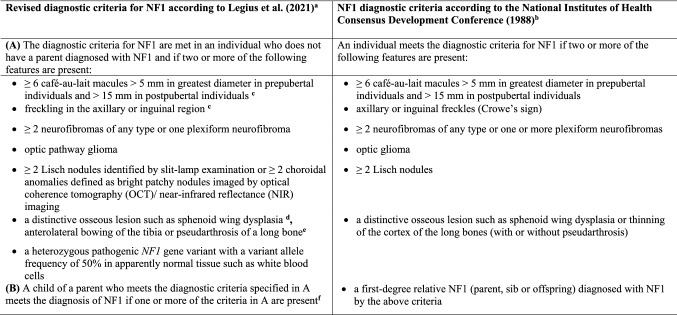
Comparison of the NIH diagnostic clinical criteria for NF1 from 1988 with the newly revised NF1 diagnostic criteria according to Legius et al. (2021)

^a^Legius et al. (2021) Revised diagnostic criteria for neurofibromatosis type 1 and Legius syndrome: an international consensus recommendation. Genet Med 23:1506–1513

^b^Neurofibromatosis: conference statement: National Institutes of Health Consensus Development Conference (1988) Arch Neurol 45:575–578

^c^If only café-au-lait macules and freckles are present, the diagnosis is most likely NF1 but exceptionally the individual might have another diagnosis such as *Legius syndrome*. At least one of the two pigmentary findings (café-au-lait macules or freckles) must be bilateral

^d^Sphenoid wing dysplasia is not a separate criterion in case of an ipsilateral orbital plexiform neurofibroma

^e^Thinning of the long bone cortex turned out not to be the primary lesion. Instead, anterolateral bowing of the lower limb and medullary canal narrowing as well as cortical thickening in the tibia and/or fibula is observed. Therefore, the orthopaedic criterion has been rephrased accordingly

^f^In the revised diagnostic criteria, only an affected parent but not affected siblings and offspring qualify as a criterion for NF1. If only siblings are affected, the diagnosis of CMMRD is possible. ‘Offspring’ was omitted because if an adult person has only one diagnostic criterion and one offspring meeting the diagnostic criteria, mosaic NF1 cannot be excluded

In this review, the challenges presented by the diagnosis of NF1 in young children are outlined, and the utility of the recently revised diagnostic criteria for NF1 (which include genetic testing) for overcoming these problems, are discussed.

## Challenges in the diagnosis of NF1 in young children and how the revised diagnostic criteria can help

The vast majority of adult patients with NF1 meet the 1988 clinical diagnostic criteria listed in Table 1. The diagnosis of NF1 may however be more difficult in infants or toddlers if they present with isolated CALM and have no family history of the disease. Many NF1-associated features are age-dependent. The first symptoms are generally CALM which may be present at birth or occur in the first year of life, often in the absence of other disease features. In the first year of life, multiple CALM are present in at least 82% of infants later diagnosed with NF1 (Huson et al. 1989). After CALM, the second most common feature in NF1 is axillary and/or inguinal freckling (Korf 1992; Nunley et al. 2009). However, axillary or inguinal freckles usually occur in children older than 2–3 years of age (Huson et al. 1989; Cnossen et al. 1998). Lisch nodules are present in the majority of adults with NF1, but only 5% of children younger than 3 years possess them (Lubs et al. 1991). Dermal neurofibromas frequently appear during puberty or young adulthood but are only very rarely detected in children younger than 3 years of age. Of the children with isolated CALM at initial presentation who are eventually diagnosed as having NF1, 76% meet the clinical NIH NF1 criteria by the age of 4 years, whereas 94% meet the criteria by the age of 6 years, and 97–100% by the age of 8 years (DeBella et al. 2000; Nunley et al. 2009). However, approximately 46% of sporadic NF1 cases fail to meet the 1988 clinical diagnostic criteria by the age of 1 year (DeBella et al. 2000). Only by the age of 6–8 years do most children with NF1 meet the 1988 clinical diagnosis criteria (DeBella et al. 2000; Nunley et al. 2009). Until this age, a definitive diagnosis of NF1 may not be possible, a shortcoming that may well impede treatment and risk stratification. Earlier diagnosis of NF1 may be beneficial to both the affected children and their families. Genetic counselling could be offered to parents and other relatives earlier, and therapeutic interventions for learning disabilities, developmental problems or other complications could be initiated sooner (Cnossen et al. 1997).

Another problem is that the 1988 clinical diagnostic criteria for NF1 are not specific if children exhibit only CALM and skin-fold freckling. Other conditions such as Legius syndrome (MIM#611431) or constitutional mismatch repair deficiency (CMMRD, MIM#276300) are also associated with these clinical manifestations in children (Brems et al. 2007; Wimmer et al. 2017; Suerink et al. 2019; Perez-Valencia et al. 2020). These disorders are caused by pathogenic variants in genes other than *NF1* and are associated with quite different disease courses. Legius syndrome is caused by pathogenic variants of the *SPRED1* gene whereas CMMRD is caused by pathogenic variants in one of four mismatch repair genes, namely *MLH1*, *MSH2*, *MSH6* or *PMS2* (Brems et al. 2007; Wimmer et al. 2017).

In the following, the most frequent and less frequent NF1-associated features in young children at risk of having NF1 are presented in greater detail.

## Frequent diagnostic features in children with NF1

### Café-au-lait macules (CALM)

One or 2 CALM occur in 2.5% of infants (< 1 year old) without a known underlying disorder (Alper et al. 1979). However, the presence of more than 3 CALM is rare, being detected in only 0.4% of healthy children ≤ 11 years of age (Whitehouse 1966; Alper et al. 1979; Burwell et al. 1982) (Table 2). NF1 is the most frequent disorder associated with the occurrence of more than 5 CALM (Lalor et al. 2020).

**Table 2:** Number of café-au-lait macules (CALM) in infants (< 1 year) and older children without a known underlying disorder as determined in 3 different studies

	Alper et al. (1979)	Whitehouse (1966)	Burwell et al. (1982)	
Age of children analysed	Newborn–1 year	1 Month–5 years	4–11 years	
Total number of children	4641	365	542	Σ = 5548
Children with 1 CALM	88 (1.9%)	69 (18.9%)	146 (26.9%)	Σ = 303 (5.5%)
Children with 2 CALM	26 (0.6%)	15 (4.1%)	30 (5.5%)	Σ = 71 (1.3%)
Children with 1 or 2 CALM	114 (2.5%)	84 (23%)	176 (32%)	Σ = 374 (6.7%)
Children with ≥ 3 CALM	10 (0.2%)	1 (0.3%)	11 (2%)	Σ = 22 (0.4%)

In children presenting with isolated CALM at the time of first investigation, the predictive value of CALM for the diagnosis of NF1 has been assessed in several studies (DeBella et al. 2000; Nunley et al. 2009; Ben-Shachar et al. 2017). These analyses indicated that the higher the number of CALM, the more likely it is that a child has indeed NF1. According to the study of Ben-Shachar et al. (2017), 79% of children younger than 14 months with ≥ 6 isolated CALM and no other disease features were diagnosed with NF1 later in life as additional clinical features of NF1 became apparent. If comprehensive genetic testing for pathogenic *NF1* variants was applied, 88.4% of children younger than 14 months with ≥ 6 isolated CALM were diagnosed with NF1 (Ben-Shachar et al. 2017). By contrast, none of the children younger than 14 months of age with fewer than 6 isolated CALM were diagnosed with NF1, at least according to the clinical diagnostic criteria.  However, 16% of these children were diagnosed with NF1 by means of genetic testing (Ben-Shachar et al. 2017). These findings indicate two important points: First, the presence of ≥ 6 CALM greatly increases the likelihood that children younger than 14 months have NF1. The risk for children with fewer than 6 CALM appears to be significantly lower, as confirmed by other studies (Korf 1992; Nunley et al. 2009). Further, these findings indicate that comprehensive genetic testing for pathogenic *NF1* variants increases the proportion of children presenting with isolated CALM, who can be diagnosed with NF1, by at least 10–16%. Thus, including the detection of a pathogenic *NF1* variant among the revised diagnostic criteria has been very important in facilitating the early diagnosis of NF1 in this subgroup of children (Legius et al. 2021) (Table 1). This conclusion has been confirmed by the results of the studies of Guigliano et al. (2019) and Castellanos et al. (2020) as summarized in Table 3.

**Table 3:** Studies that have investigated children with isolated CALM but no other NF1-associated disease features and without an affected first-degree relative by means of comprehensive genetic testing of the *NF1* gene (and the *SPRED1* gene)

Total number of patients investigated	Age of the patients	Number of CALM	Number of patients with pathogenic variants in the *NF1* gene	Number of patients with pathogenic variants in the *SPRED1* gene	Number of patients without a pathogenic variant	References
44	≤ 9 Years	≥ 6	28 (63.6%)	1 (2.3%)	15 (34.1%)	Giugliano et al. (2019)
71	0–7 Years (*N* = 42); > 7 Years (*N* = 8); age unknown (*N* = 21)	≥ 6	34 (47.9%)^a^	3 (4.2%)^b^	25 (35.2%)	Castellanos et al. (2020)
95	≤ 14 Months	≥ 6	84 (88.4%)	Not investigated	11 (11.6%)	Ben-Shachar et al. (2017)^c^
65	> 14 Months and ≤ 29 months	≥ 6	45 (69.2%)	Not investigated	20 (30.8%)	Ben-Shachar et al. (2017)^c^
38	≤ 14 Months	< 6	6 (15.8%)	Not investigated	32 (84.2%)	Ben-Shachar et al. (2017)^c^

^a^In addition to 34 pathogenic *NF1* variants, 7 patients with missense *NF1* variants of unknown significance (VUS) were identified. Thus, the total number of *NF1* variants identified was *N* = 41 (57.8%)

^b^In addition to 3 pathogenic *SPRED1* variants, two patients with *SPRED1* variants of unknown significance (VUS) were identified. Thus, the total number of *SPRED1* variants identified was *N* = 5 (7%)

^c^In this study, patients under suspicion of segmental NF1 were excluded but children were not tested for possible generalized mosaic NF1

In addition to the number of CALM, the age of the child may also be an important factor in terms of a putative diagnosis of NF1. None of 50 children with fewer than 6 CALM who were 29 months or older were given a diagnosis of NF1 on the grounds that they did not have a pathogenic *NF1* variant or did not meet the 1988 clinical diagnostic criteria (Ben-Shachar et al. 2017).

In addition to the number of CALM, their morphological appearance is also of some predictive value in determining whether a child is at higher or lower risk of having NF1. Typical CALM present with homogeneous pigmentation and regular borders. By contrast, atypical CALM exhibit irregular margins and ragged borders. Children who were 29 months or older with atypical CALM had a very low risk of having molecularly or clinically confirmed NF1 (Ben-Shachar et al. 2017). Furthermore, patients with NF1 who were found to harbour pathogenic *NF1* variants had atypical CALM six-fold less frequently than individuals without pathogenic *NF1* variants (Ben-Shachar et al. 2017). This concurs with the results of Nunley et al. (2009) who observed that 47% of children with isolated typical CALM were diagnosed as having NF1 later in life by means of clinical criteria, whereas only 5% of children with atypical CALM developed NF1. Comprehensive genetic testing for pathogenic *NF1* variants, as included now in the revised criteria for NF1, may facilitate the diagnosis in patients with atypical CALM in the absence of further NF1-associated disease features.

### Skinfold freckling

Axillary and inguinal freckles are generally 1–3 mm in diameter and appear as tiny brown spots, often in groups. In contrast to CALM, these freckles become apparent later in childhood. The youngest patients exhibiting freckles are 2–3 years old (Huson et al. 1989; Cnossen et al. 1998). In children suspected of having NF1 on the grounds that they exhibit ≥ 6 isolated CALM at initial presentation, the second most common disease feature used to establish the diagnosis of NF1 is axillary and/or inguinal freckling, occurring in 77% of patients (Nunley et al. 2009). By the age of 6 years, 81% of patients with NF1 exhibit freckling and by the age of 7 years almost 90% have freckling (Obringer et al. 1989; DeBella et al. 2000). Since freckles usually become visible only after the age of 2–3 years, they do not facilitate the diagnosis in younger children presenting with isolated CALM and no family history of NF1. Until the appearance of skin-fold freckling in this group of children, a definitive diagnosis of NF1 is not possible without molecular testing for a pathogenic *NF1* variant.

### Lisch nodules

Lisch nodules are benign hamartomas of the iris. Isolated Lisch nodules are very rarely seen in individuals from the general population (reviewed by Cassiman et al. 2013). By contrast, the vast majority of adult NF1 patients have multiple Lisch nodules (Huson et al. 1989; Lubs et al. 1991). Hence, Lisch nodules are a highly specific diagnostic criterion (Lubs et al. 1991). It has been shown that light irides harbour significantly more Lisch nodules than dark irides what may be explicable in terms of the photo-protective effects of pigmentation (Boley et al. 2009). Furthermore, Lisch nodules are primarily located in the inferior hemifield (half) of the iris, irrespective of its colour. These findings suggest that UV radiation and DNA damage may play a role in the pathogenesis of Lisch nodules (Boley et al. 2009). Although Lisch nodules appear during childhood, only 5% of children younger than 3 years have them (Lubs et al. 1991). The estimated prevalence of Lisch nodules is 42% among children with NF1 who are 3–5 years old and 55% in children aged 5–6 years (Lubs et al. 1991). Another study has reported Lisch nodules in 52% of children with NF1; the mean age at presentation of Lisch nodules in these patients was 8.8 years with a standard deviation of 3.6 years (Cnossen et al. 1998). Thus, Lisch nodules are an important diagnostic feature of NF1 in older children but may not allow for a definitive diagnosis of NF1 in infants with multiple CALM. Lisch nodules are not however observed in patients with Legius syndrome and hence may facilitate the differential diagnosis by means of clinical criteria.

### Choroidal anomalies

Fundus examination by means of near-infrared reflectance with a scanning laser ophthalmoscope (NIR) and ocular coherence tomography (OCT) have indicated that choroidal anomalies are frequent in patients with NF1 (Yasunari et al. 2000; Viola et al. 2012; Goktas et al. 2014; Parrozzani et al. 2015; Vagge et al. 2015; Cassiman et al. 2017; Tucci et al. 2017). Choroidal anomalies appear as ovoid, bright patches or nodules consisting of proliferating Schwann cells and melanocytes arranged in concentric rings around axons (Viola et al. 2012). Choroidal involvement has previously been considered to be a rare finding in NF1, due to the fact that these lesions are asymptomatic and not detectable by conventional ophthalmoscopy or fluorescein angiography.

The presence and number of choroidal anomalies (CA) is age-dependent; their prevalence is lower in children with NF1 than in adults with the disease. Whilst CA are observed in 86–100% of adult NF1 patients (Yasunari et al. 2000; Viola et al. 2012), the prevalence of CA in children with NF1 younger than 12 years is 60.5–78.9% (Viola et al. 2012; Goktas et al. 2014; Parrozzani et al. 2015; Vagge et al. 2015).

CA appear to be as frequent as, or even more frequent than, Lisch nodules in children with NF1 (Viola et al. 2012; Parrozzani et al. 2015). Most importantly, CA can be detected in children as young as 2 years of age (Vagge et al. 2015). By contrast, in many children with NF1, Lisch nodules appear only later, around the age of 3–5 years (Lubs et al. 1991).

CA are only rarely detected or not all in healthy controls (Viola et al. 2012; Goktas et al. 2014; Parrozzani et al. 2015; Vagge et al. 2015; Cassiman et al. 2017). If CA are present in healthy controls, they represent single lesions (Cassiman et al. 2017). In NF1 patients, CA are generally present as multiple lesions; 56% of patients exhibit more than 4 CA (Cassiman et al. 2017). Multiple CA are not detected in patients with Legius syndrome (Cassiman et al. 2017; Tucci et al. 2017) and only two of 19 patients with Legius syndrome were found to have a single choroidal anomaly (Cassiman et al. 2017; Tucci et al. 2017). These findings imply that CA are very rare in patients with Legius syndrome and may represent an important distinguishing diagnostic feature. Further, since CA are present at an early stage, they may facilitate the diagnosis of NF1 in children with isolated CALM who do not meet the 1988 clinical diagnostic criteria. Therefore, CA have been included in the revised criteria for the diagnosis of NF1 (Table 1) (Legius et al. 2021). The NIR method is non-invasive and generally well tolerated by children. However, the success of the investigation is very much dependent upon the degree of cooperation given by the child. Parrozzani et al. (2015) investigated 160 children with a mean age of 8 years (range 2–13 years) by means of NIR and the feasibility rate was found to be 82%. Of these 160 children, 119 were already diagnosed with NF1 since they met the clinical diagnostic criteria and 72 (60.5%) of them had CA. The remaining 41 children were suspected to have NF1 although they did not meet the NIH criteria. One of them was a 2-year old boy with more than 5 CALM but no other symptoms of NF1. Since this boy had CA, he was diagnosed with NF1. Likewise, Vagge et al. (2015) detected CA in a 2-year old child, indicative of the early appearance of this diagnostic feature of NF1. So far, there are no indications for the assumption that CA would cause clinical complications during the course of the disease. Since CA have a high specificity and sensitivity for NF1, they have been added as an ophthalmic criterion to the revised diagnostic criteria for NF1 (Legius et al. 2021) (Table 1).

### Plexiform neurofibromas and optic pathway gliomas

Plexiform neurofibromas (PNFs) are congenital lesions characterized by tumour cells that spread along multiple fascicles of the nerve, leading to a diffuse mass of thickened nerve fibres embedded within a proteinaceous matrix. PNFs may be located superficially and/or internally and hence only detectable by MRI scans, particularly in young children. The estimated prevalence of externally visible or palpable plexiform neurofibromas (but not internal PNF) in children with NF1 is 26.6–30% (Huson et al. 1989; Cnossen et al. 1998; Prada et al. 2012). Whole-body MRI of 65 children and adolescents with NF1 aged between 1.7 and 17.6 years indicated that 37 (57%) had internal PNF which were asymptomatic in 20 (31%) of the patients (Nguyen et al. 2011). In approximately 50% of all children with PNF, the age at clinical identification of the tumours is 0–3 years (Prada et al. 2012). Since PNFs occur in the vast majority of cases in the context of NF1, they are a rather specific diagnostic criterion for NF1.

Optic pathway gliomas (OPGs) are low grade astrocytomas observed in 14–20% of patients with NF1 as determined by neuroimaging (Listernick et al. 1994; Blanchard et al. 2016; Friedrich and Nuding, 2016). NF1-associated OPGs are most often diagnosed during childhood. Children ≤ 6 years of age have the highest risk to develop a symptomatic OPG (Listernick et al. 1994). The mean age at diagnosis of a symptomatic OPG was 4.2 years (Listernick et al. 1994), 4.6 years (Trevisson et al. 2017), 5.1 years (Nicolin et al. 2009) and 7.6 years (Friedrich and Nuding 2016). Asymptomatic OPGs are often diagnosed later in life, with a mean age at diagnosis of 5.3 years (Listernick et al. 1994), 5.9 years (Trevisson et al. 2017), 11.6 years (Friedrich and Nuding 2016). Visual acuity, strabismus, exophthalmus and proptosis are the most common symptoms caused by these tumours (Listernick et al. 2007; Friedrich and Nuding 2016). A proportion of OPGs are already present at birth and their detection in young children by MRI may facilitate the diagnosis of NF1. Taken together, PNFs and optic pathway gliomas are important diagnostic features in young children with NF1.

## Less common disease features in children with NF1

### Long-bone dysplasia

Long-bone dysplasia, seen in 5% of patients with NF1 typically involves the tibia and frequently presents with anterolateral bowing that may progress to fracture (Riccardi 1981). In addition to the tibia, fibula, radius and ulna are also potential sites of dysplasia even though less commonly. Long-bone dysplasia is most often unilateral, evident in the first year of life, and usually not associated with a neurofibroma (Stevenson et al. 1999). Congenital anterolaterally bowed tibia without pseudarthrosis is observed in 3–4% of young children with NF1 (Friedman and Birch 1997). It may be apparent at birth but in most cases, it is recognized months later albeit within the first year of life. Remarkably, tibial dysplasia with pseudarthrosis is caused by double inactivation of the *NF1* gene (Stevenson et al. 2006; Sant et al. 2015). Thus, these osseous lesions as well as other frequent clinical symptoms in NF1 such as CALM, neurofibromas and other tumours arise according to the two-hit model of tumorigenesis (Serra et al. 1997, 2000; Maertens et al. 2007; De Schepper et al. 2008; reviewed by Brems et al. 2009). The prevalence of patients with NF1 among patients with congenital arthrosis of the tibia has been estimated to be 85% (Van Royen et al. 2016). Germline *NF1* pathogenic variants are present in patients with NF1 but are absent in patients with congenital pseudarthrosis of the tibia without NF1 thereby differentiating these patient groups (Zhu et al. 2019).

The NIH diagnostic criteria for NF1 from 1988 included the criterion: “thinning of the long bone cortex with or without pseudarthrosis” (Table 1). However, thinning of the long bone cortex turned out not to be the primary lesion (Stevenson et al. 2007). Instead, anterolateral bowing of the lower limb and medullary canal narrowing as well as cortical thickening in the tibia and/or fibula is observed (Stevenson et al. 2007). In the revised version of the diagnostic criteria, the orthopaedic criterion has been rephrased accordingly (Table 1).

### Sphenoid wing dysplasia

Unilateral dysplasia of the greater wing of the sphenoid bone is one of the most distinctive craniofacial lesions in NF1, being observed in 3–11% of cases (reviewed by Alwan et al. 2005). Sphenoid wing dysplasia (SWD) is congenital but becomes clinically apparent later in life, frequently before the age of 2 years. SWD can be asymptomatic and is diagnosed by skull radiographs or CT scans. SWD is relatively rare in the general population, and over 50% of cases are associated with NF1 (reviewed by Chauvel-Picard et al. 2020). SWD may become progressive and cause disruption of the orbit and consequent pulsating exophthalmos. Abnormal growth of the skull associated with sphenoid wing lesions in children with NF1 may also lead to progressive facial deformities (Jacquemin et al. 2002). SWP is thought to result from a primary ossification defect with poor mesodermal development and bone formation (reviewed by Alwan et al. 2005; Chauvel-Picard et al. 2020). However, SWP may also occur concurrent with orbital-periorbital plexiform neurofibromas (reviewed by Avery et al. 2017). According to the newly revised diagnostic criteria for NF1, sphenoid wing dysplasia is not a separate diagnostic criterion in case of an ipsilateral orbital plexiform neurofibroma (Table 1).

## Genetic testing in NF1

New in the revised version of the diagnostic criteria is the inclusion of the detection of a pathogenic *NF1* gene variant as a separate diagnostic criterion (Legius et al. 2021).

Variants identified in the *NF1* gene are classified as pathogenic according to the guidelines developed by the American College of Medical Genetics and Genomics, the Association for Molecular Pathology and the College of American Pathologists (Richards et al. 2015). The *NF1* gene is relatively large, encompassing ~ 350 kb and 55 constitutive exons as well as five alternatively spliced exons. *NF1* encodes neurofibromin, a multifunctional protein with at least 6 different functional domains involved in the regulation of various signalling pathways (reviewed by Bergoug et al. 2020). More than 3600 different pathogenic *NF1* variants have now been reported by The Human Gene Mutation Database (HGMD; http://www.hgmd.org/); these are located across the gene coding regions as well as within the introns thereby interfering with the splicing process (Stenson et al. 2020). Whilst some 31 different pathogenic *NF1* variants exhibit a prevalence of ≥ 0.5% among NF1 patients, it has been estimated that approximately 46% of NF1 patients carry extremely rare or private pathogenic *NF1* gene variants (Koczkowska et al. 2018, 2019, 2020). In addition, copy number variants of single and multiple *NF1* exons have been identified, as well as deletions spanning the entire *NF1* gene which are observed in 5–11% of all NF1 patients (Messiaen, 2020; Kehrer-Sawatzki and Cooper 2021).

Genetic testing in NF1 is complicated by the large size of the gene with its long stretches of intronic DNA, the extensive allelic heterogeneity (i.e. the large number of different pathogenic and likely pathogenic *NF1* variants) and the challenging interpretation of potentially pathogenic *NF1* variants, particularly missense and in-frame deletion/insertion variants. However, a comprehensive genetic testing protocol can ensure a high detection rate of the pathogenic variants residing within or involving the *NF1* gene as well the accurate and reliable interpretation of their molecular consequences (Messiaen et al. 2000; Wimmer et al. 2006; Pros et al. 2008; Valero et al. 2011; Sabbagh et al. 2013; Imbard et al. 2015; Pasmant et al. 2015; Evans et al. 2016; Pasmant and Vidaud 2016; Messiaen 2020). Such a protocol should include RNA analysis by means of cDNA sequencing, the sequence analysis of the coding sequence and intron/exon boundaries of the *NF1* gene, as well as the copy number analysis of *NF1* exons and whole gene deletions (Messiaen et al. 2000; Wimmer et al. 2006; Pros et al. 2008; Valero et al. 2011; Sabbagh et al. 2013; Pasmant et al. 2015; Evans et al. 2016; Pasmant and Vidaud 2016; Giugliano et al. 2019; Castellanos et al. 2020; Messiaen 2020). By means of a comprehensive mutation testing protocol in 361 NF1 patients with more than pigmentary diagnostic criteria, a potentially pathogenic *NF1* variant was identified in 166/171 (97%) of familial cases and 182/190 (96%) of sporadic cases (Evans et al. 2016). If class 3 variants of unknown pathogenicity according to Plon et al. (2008) and Richards et al. (2015) had not been included, the pathogenic variant detection rate decreased to 154/171 (90%) in familial cases and 175/190 (92%) in sporadic cases. Similarly high mutation detection rates have been obtained in other studies that employed comprehensive genetic testing of the *NF1* gene (Messiaen et al. 2000; Valero et al. 2011; Sabbagh et al. 2013; Guigliano et al. 2019). cDNA sequencing is superior to standard exon sequencing since a broader spectrum of pathogenic variants can be identified, including deep intronic variants. Further, it improves the detection of those pathogenic missense variants that affect splicing (Messiaen et al. 2000; Evans et al. 2016; Pasmant and Vidaud, 2016).

The recruitment of genetic testing to make possible a definitive diagnosis of NF1 is particularly important in children and young adults with isolated multiple CALM but without a family history of the disease (Tsang et al. 2012; Yao et al. 2016). This assertion is supported by the study of Evans et al. (2016) who analysed 71 individuals with ≥ 6 CALM with or without freckling, but no other NF1 diagnostic criterion, who also lacked a parent with any NF1 criterion and were < 20 years of age. Of these 71 individuals, 44 (62%) harboured a clearly pathogenic *NF1* variant, 3 had *NF1* variants of uncertain pathogenicity, 6 (8.5%) a pathogenic *SPRED1* variant whilst 18 (25.3%) did not possess a disease-causing variant in either *SPRED1* or *NF1*. Thus, the likelihood that a child with ≥ 6 CALM with or without freckling and no other NF1 criterion has non-mosaic NF1 is 62–66%. This conclusion is in accord with the results of other studies that determined the pathogenic *NF1* variant detection rate in children with isolated multiple CALM and no family history of the disease (Table 3). A negative testing result for a pathogenic *NF1* variant reduces the likelihood to have constitutional (non-mosaic) NF1 to ~ 11% in children and young adults with ≥ 6 CALM with or without freckling, but no other NF1 diagnostic criterion (Evans et al. 2016).

Genetic testing performed to detect pathogenic *NF1* variants may also facilitate the diagnosis in some families with spinal NF1 (Burkitt Wright et al. 2013) or patients with the pathogenic in-frame deletion in *NF1* exon 17 at position c.2970-2972 delAAT (p.Met992del) which is associated with typical pigmentary features of NF1, but not with neurofibromas (Upadhyaya et al. 2007; Quintans et al. 2011; Koczkowska et al. 2019). Likewise, patients with various different *NF1* missense pathogenic variants at amino acid residue Arg1809 have multiple CALM with or without freckling and rarely Lisch nodules, but do not exhibit neurofibromas (Pinna et al. 2015; Rojnueangnit et al. 2015; Santoro et al. 2015). In these cases, the genetic testing isn’t simply to facilitate the clinical diagnosis, but also to allow a prediction to be made about the clinical phenotype from the mutant genotype detected. NF1 patients with these lesions may not meet the 1988 clinical diagnostic criteria, particularly during childhood. However, they would do so if the 2021 criteria would be applied including genetic testing. By contrast, 95% of individuals with classic NF1 fulfil two or more of the 1988 clinical diagnostic criteria (not taking family history into account) by ≥ 9 years of age (DeBella et al. 2000). Individuals with p.Arg1809 or p.Met992del pathogenic variants are less likely than NF1 patients of the same age who harbour other *NF1* variants to fulfil the 1988 clinical criteria at ≥ 9 years of age (Rojnueangnit et al. 2015). In the same vein, pathogenic missense variants affecting *NF1* residue Met1149 are associated with a mild phenotype characterized by the lack of NF1-associated tumours (Koczkowska et al. 2020). These authors reported that 16.7% of patients with pathogenic missense variants affecting residue Met1149 who were older than 9 years did not fulfil the 1988 clinical diagnostic criteria (Koczkowska et al. 2020). In these families, genetic testing is therefore important to confirm the clinical diagnosis and identify at-risk relatives (Jett and Friedman 2010).

Even though genetic testing may facilitate the diagnosis of NF1 in young oligosymptomatic children without a family history of the disease, some parents and physicians may object to genetic testing for practical or financial reasons and might instead opt to wait until further diagnostic symptoms may (or may not) appear.

## Differential diagnosis of NF1 versus Legius syndrome and CMMRD

Differential diagnosis is particularly difficult in children with multiple isolated CALM without a family member with multiple CALM, NF1 or Legius syndrome. These children may not be affected by NF1 but instead by Legius syndrome or less frequently by another RASopathy such as Noonan syndrome with multiple lentigines (formerly known as LEOPARD syndrome, MIM#151100) or Constitutive mismatch repair deficiency (CMMRD) (Brems et al. 2007; Shah et al. 2010; Santoro et al. 2014; Santos et al. 2016; Wimmer et al. 2017; Suerink et al. 2019; Anderson 2020; Jha et al. 2020; Perez-Valencia et al. 2020).

NF1 and Legius syndrome are the RASopathies with the most pronounced overlap in clinical symptoms. RASopathies constitute a group of genetic disorders that are caused by germline pathogenic variants affecting RAS-mitogen activated protein kinase (MAPK) pathway genes leading to RAS/MAPK pathway dysregulation (reviewed by Rauen 2013). To improve the differential diagnosis of NF1, the detection of pathogenic variants in the *NF1* gene has been included among the revised diagnostic criteria for NF1 (Table 1) (Legius et al. 2021). In children with multiple isolated CALM and without affected family members, genetic testing indicated a detection rate of pathogenic *NF1* variants in the range of 48% up to 88%, depending on the age of the children and the number of CALM (Ben-Shachar et al. 2017; Giugliano et al. 2019; Castellanos et al. 2020) (Table 3).

### Legius syndrome

Legius syndrome (MIM#611431) is characterized by the occurrence of CALM with or without axillary or inguinal freckles and therefore resembles NF1. Learning disabilities, attention deficits and hyperactivity may also occur in patients with Legius syndrome even though they are generally less frequent and less severe than in NF1 (Brems and Legius 2013; Denayer and Legius 2020). Other NF1-associated features such as Lisch nodules, neurofibromas, NF1-specific bone lesions, optic pathway gliomas, and malignant peripheral nerve sheath tumours are however absent in patients with Legius syndrome (Brems et al. 2007, 2012; Messiaen et al. 2009; Pasmant et al. 2009; Spurlock et al. 2009; Laycock-van Spyk et al. 2011; Brems and Legius 2013; Denayer and Legius 2020).

Legius syndrome has been estimated to occur with a prevalence of 1:46,000–1:75,000 and is caused by pathogenic variants in the *SPRED1* gene located on chromosome 15q13.2 (Brems et al. 2007, 2012; Messiaen et al. 2009; Pasmant et al. 2009; Spurlock et al 2009; Laycock-van Spyk et al. 2011). The mode of inheritance is autosomal dominant and 65% of patients with Legius syndrome have a family history of the disease. *SPRED1* encodes the sprouty-related EVH1 domain–containing protein 1. Via its EVH1 domain, the SPRED1 protein binds to neurofibromin, the protein product of the *NF1* gene, thereby recruiting it to the plasma membrane. Neurofibromin is a negative regulator of RAS and its interaction with SPRED1 leads to the down-regulation of the RAS-MAPK signal transduction pathway (Wakioka et al. 2001; Stowe et al. 2012; Dunzendorfer-Matt et al. 2016; Lorenzo and McCormick 2020).

In young children presenting with CALM with or without freckling and no other NF1-associated features or a family history of the disease, the differential diagnosis between NF1 and Legius syndrome is not possible by means of the 1988 clinical diagnostic criteria. However, an early differential diagnosis is very important in terms of patient care and risk stratification since Legius syndrome is not associated with the tumour phenotype seen in NF1.

In families with an autosomal dominant phenotype of CALM with or without freckles but without other NF1-associated disease features or a pathogenic *NF1* gene variant, 19% have a mutation in the *SPRED1* gene (Messiaen et al. 2009; Brems et al. 2012). It has been estimated that 1–8.5% of patients with multiple CALM with or without freckling but no other NF1 diagnostic features, have Legius syndrome (Evans et al. 2016; Brems and Legius, 2013; Pasmant et al. 2015; Bernier et al. 2016).

Approximately 50% of patients with Legius syndrome meet the 1988 clinical diagnostic criteria for NF1 since they have ≥ 6 CALM with freckles or have ≥ 6 CALM without freckles and a family member with CALM and freckles (Messiaen et al. 2009). Approximately 1–4% of patients with multiple CALM who meet the 1988 clinical diagnostic criteria for NF1 have Legius syndrome since they harbour *SPRED1* pathogenic variants (Brems et al. 2012). Consequently, the diagnostic criteria for NF1 had to be revised (Legius et al. 2021). Concomitantly, the diagnostic criteria for Legius syndrome were formulated for the first time by an international board of experts (Table 4) (Legius et al. 2021). It was most important to be able to distinguish between the two conditions since they differ markedly in terms of the severity of their clinical manifestations. In contrast to NF1, frequent surveillance for tumours is not necessary in children and adults with Legius syndrome (Denayer and Legius 2020).

**Table 4:** Diagnostic criteria for Legius syndrome according to Legius et al. (2021)

(A) The diagnostic criteria for Legius syndrome are met in an individual who does not have a parent diagnosed with Legius syndrome if both of the following criteria are present:
≥ 6 Café-au-lait macules bilaterally distributed and no other NF1-related diagnostic criteria except for axillary or inguinal freckling^a^
A heterozygous pathogenic variant in *SPRED1* with a variant allele fraction of 50% in an apparently normal tissue such as white blood cells
(B) A child of a parent who meets the diagnostic criteria specified in (A) merits a diagnosis of Legius syndrome if one or more of the criteria in (A) are present

^a^The presence of fewer than 6 café-au-lait macules does not exclude Legius syndrome

It is important to add that in some patients with multiple isolated CALM, pathogenic variants are not found in either the *SPRED1* gene or the *NF1* gene by the molecular testing of blood. In sporadic patients, the underlying cause may be somatic mosaicism for a pathogenic *NF1* or *SPRED1* variant that is present only in melanocytes and absent from blood cells (Maertens et al. 2007; Legius et al. 2021). To provide guidance for the correct diagnosis, diagnostic criteria for mosaic NF1 and mosaic Legius syndrome have also been formulated and published as an international consensus recommendation (Legius et al. 2021). It has been estimated that ~ 10% of sporadic NF1 patients have mosaic NF1 caused by postzygotic *NF1* mutations that are absent from, or present in, a very low proportion of blood lymphocytes (Messiaen et al. 2000). This may be concluded from the pathogenic variant detection rate determined by means of comprehensive genetic testing including the genomic DNA and cDNA sequencing of the *NF1* gene as well as the assessment of copy number variants of single and multiple *NF1* exons and entire *NF1* gene deletions (Messiaen et al. 2000). Mosaicism has also been observed in Legius syndrome but it would appear to be rare (Jobling et al. 2017). The clinical suspicion of mosaicism justifies genetic testing for Legius syndrome and NF1, as now included in the newly formulated diagnostic criteria for the mosaic forms of both conditions (Legius et al. 2021).

### CMMRD

Constitutive mismatch repair deficiency (CMMRD, MIM#276300) is a rare autosomal recessive inherited disease caused by pathogenic variants in one of the mismatch repair genes *MLH1*, *MSH2*, *MSH6* or *PMS2* (Bakry et al. 2014; Wimmer et al. 2014, 2017; Suerink et al. 2019; Perez-Valencia et al. 2020; Aronson et al. 2021; Duorno et al. 2021). Children with CMMRD often have multiple CALM that are similar to those seen in patients with NF1 or Legius syndrome. Hence, CMMRD should be considered when arriving at a differential diagnosis of children presenting with multiple CALM. Some patients with CMMRD even meet the clinical NIH diagnostic criteria for NF1 (Wimmer et al. 2014). It is important to distinguish between these conditions because CMMRD is associated with a high risk of childhood malignancy unlike NF1 and Legius syndrome (Bakry et al. 2014; Wimmer et al. 2014, 2017; Suerink et al. 2019; Perez-Valencia et al. 2020; Aronson et al. 2021; Duorno et al. 2021).

The occurrence of CALM in patients with CMMRD is intriguing. Most likely, CALM (and other *NF1*-associated symptoms such as neurofibromas or freckling) in patients with CMMRD are caused by postzygotic *NF1* mutations. This has been concluded from the observation that several patients with CMMRD exhibited a segmental distribution of these lesions and from the detection of postzygotic *NF1* variants causing somatic mosaicism in CMMRD patients (Wang et al. 1999; Auclair et al. 2007; Alotaibi et al. 2008). The large size of the *NF1* gene and its high mutation rate, indicated by the fact that at least 50% of all NF1 cases are sporadic, may render the *NF1* gene highly susceptible to postzygotic mutations in the absence of MMR activity (Wang et al. 1999). The need for a differential diagnosis to distinguish between CMMRD, NF1 and Legius syndrome as well as mosaic NF1 has been emphasised in the revised diagnostic criteria for NF1 (Legius et al. 2021).

The 1988 NIH clinical diagnostic criteria for NF1 included the criterion “a first-degree relative NF1 (parent, sib or offspring) diagnosed with NF1” (Table 1). This criterion has been restricted to “parent”, and “sib or offspring” has been removed from the revised criteria by Legius et al. (2021) (Table 1). An affected sibling no longer qualifies as a criterion for NF1 since if only siblings are affected, a diagnosis of CMMRD would also be possible. Additionally, having affected offspring no longer qualifies as a diagnostic criterion for constitutional NF1. If an adult person only has one NF1 criterion in addition to an affected child fulfilling the diagnostic criteria, this person is likely to have mosaic NF which should be differentiated from constitutional NF1. Legius et al. (2021) stated that the revised criterion, which only includes “parent with NF1”, is specific enough to correctly diagnose most offspring presenting with one diagnostic criterion as having NF1.

## Mosaicism

Somatic mosaicism caused by a postzygotic pathogenic *NF1* variant may be responsible for isolated CALM in patients without a family history of NF1. In general, mosaicism in NF1 may either be associated with a generalized form of the disease, with symptoms located in a disseminated manner and not restricted to a specific body part. Alternatively, mosaic NF1 can present in a segmental form characterized by symptoms such as CALM limited to one side of the body or one specific limb or segment. Mosaic NF1 is often associated with a milder disease manifestation than generalized NF1 caused by a germline pathogenic *NF1* variant and may be responsible for an oligosymptomatic disease manifestation (Ruggieri and Huson 2001; Tinschert et al. 2000; García-Romero et al. 2016; Ejerskov et al. 2021). Remarkably, many children with segmental NF1 have only localized pigmentary changes (Listernick et al. 2003; Lara-Corrales et al. 2017; Hom et al. 2020). In addition to the revised diagnostic criteria for NF1 and Legius syndrome, Legius et al. (2021) also recommended diagnostic criteria for the mosaic forms of both disorders. According to Legius et al. (2021), mosaic NF1 in a patient with clinical signs of the disease is confirmed if the patient harbours a heterozygous pathogenic *NF1* variant in unaffected tissue such as blood but in significantly fewer than 100% of cells (as indicated by a variant allele fraction of < 50%). Further, mosaic NF1 is confirmed if an identical first hit pathogenic *NF1* variant is identified in two or more anatomically unrelated lesions such as CALM or neurofibromas in the absence of this pathogenic *NF1* variant in unaffected tissue such as blood. The retrieval of biopsies taken from small children suspected of having NF1 could be regarded as problematic but has been successfully applied to diagnose small plexiform neurofibromas below irregular shaped CALM or hyperpigmented regions by histopathological analysis which served to confirm the diagnosis of NF1 in children with a mean age of 6.7 years (range: 6 months–18 years) (García-Martínez et al. 2021).

## Conclusion/summary

The revised diagnostic criteria for NF1 comprise all the clinical criteria included in the NIH diagnostic criteria from 1988 which proved to be very helpful for the diagnosis of NF1, particularly in older children and adults. Additionally, the revised diagnostic criteria include choroidal anomalies as a new ophthalmic criterion of high importance since choroidal anomalies may be observed earlier than Lisch nodules and have a high sensitivity and specificity for NF1. Furthermore, the revised diagnostic criteria for NF1 include the detection of a pathogenic variant of the *NF1* gene as a separate diagnostic criterion. Taken together, the revised diagnostic criteria for NF1 may facilitate an early diagnosis, particularly in young children with isolated CALM and without a parent with NF1. The revised diagnostic criteria for NF1, together with the newly formulated diagnostic criteria for Legius syndrome which include genetic testing for pathogenic variants in *SPRED1*, promise to facilitate the differential diagnosis of both disorders at an early age.

## Declarations

### Data availability

The manuscript does not have associated data.

### Code availability

The manuscript does not include software application or custom codes**.**
